# Supplementary material for: MetaRibo-Seq measures translation in microbiomes
Source: Nat Commun. 2020 Jun 29;11:3268. doi: 10.1038/s41467-020-17081-z (PMC7324362; doi:10.1038/s41467-020-17081-z)
Supplement: Supplementary file 10 — Supplementary Data 7 [file 41467_2020_17081_MOESM10_ESM.zip › File2/Confidence_VeryHigh_Taxonomy/83917_out.krona.html]

Javascript must be enabled to view this page.

members
magnitude
magnitudeUnassigned
count
unassigned
taxon
rank

83917\_out

7

3

SRS017745\_contig\_number\_contig-100\_24401.24402SRS1041136\_contig\_number\_contig-100\_13994.89838SRS146813\_contig\_number\_contig-100\_44396.44396

4
superkingdom
2

4
1239
phylum

4
class
186801

186802
order
4


SRS012849\_contig\_number\_contig-100\_786.167442
2
family
1
541000

genus
946234
1

292800
species
1

SRS147377\_contig\_number\_12656


SRS052697\_contig\_number\_contig-100\_381.241625SRS142503\_contig\_number\_52422
2
species
172733
